# Prioritizing candidate eQTL causal genes in *Arabidopsis* using RANDOM FORESTS

**DOI:** 10.1093/g3journal/jkac255

**Published:** 2022-09-23

**Authors:** Margi Hartanto, Asif Ahmed Sami, Dick de Ridder, Harm Nijveen

**Affiliations:** Bioinformatics Group, Wageningen University and Research, 6708 PB Wageningen, The Netherlands; Bioinformatics Group, Wageningen University and Research, 6708 PB Wageningen, The Netherlands; Bioinformatics Group, Wageningen University and Research, 6708 PB Wageningen, The Netherlands; Bioinformatics Group, Wageningen University and Research, 6708 PB Wageningen, The Netherlands

**Keywords:** *Arabidopsis thaliana*, eQTL, gene expression, machine learning, causal gene

## Abstract

Expression quantitative trait locus mapping has been widely used to study the genetic regulation of gene expression in *Arabidopsis thaliana*. As a result, a large amount of expression quantitative trait locus data has been generated for this model plant; however, only a few causal expression quantitative trait locus genes have been identified, and experimental validation is costly and laborious. A prioritization method could help speed up the identification of causal expression quantitative trait locus genes. This study extends the machine-learning-based QTG-Finder2 method for prioritizing candidate causal genes in phenotype quantitative trait loci to be used for expression quantitative trait loci by adding gene structure, protein interaction, and gene expression. Independent validation shows that the new algorithm can prioritize 16 out of 25 potential expression quantitative trait locus causal genes within the top 20% rank. Several new features are important in prioritizing causal expression quantitative trait locus genes, including the number of protein–protein interactions, unique domains, and introns. Overall, this study provides a foundation for developing computational methods to prioritize candidate expression quantitative trait locus causal genes. The prediction of all genes is available in the AraQTL workbench (https://www.bioinformatics.nl/AraQTL/) to support the identification of gene expression regulators in *Arabidopsis*.

## Introduction

One of the main objectives of genetic research is to link traits to genotypic variation. However, the path from genetics to observable traits is not straightforward; instead, it goes through a network of interconnecting intermediate phenotypes, such as gene expression, protein levels, and metabolite levels (Civelek and Lusis 2013). Studying the effect of genetic perturbation on these intermediate phenotypes could improve our understanding of how a trait is regulated. Following recent advances in omics technology, the effect of multiple genetic perturbations can now be studied in a single experiment using linkage mapping or association studies. One example is genetical genomics, where variation in transcript levels is statistically associated with genetic variation in a population (Jansen and Nap 2001) to find so-called expression quantitative trait loci (eQTLs).

A mapped eQTL can be categorized as *cis* or *trans* based on its location relative to the affected gene. *Cis*-eQTLs are mapped close to the gene and are assumed to arise due to sequence polymorphisms in or near the gene itself, for instance, in *cis*-regulatory elements (e.g. the promoter). In contrast, *trans*-eQTLs are mapped far away from the target gene and emerge due to polymorphisms in *trans*-acting factors (e.g. transcription factors) called expression quantitative trait genes or eQTGs (Brem *et al.* 2002; Rockman and Kruglyak 2006). However, a *trans*-eQTL typically spans a large genomic region with hundreds of candidate eQTGs. Experimental fine mapping to narrow down the region (e.g. in Eshed and Zamir 1995) is costly and laborious. As a result, only a few causal genes have been identified in the thousands of eQTLs that have been mapped for *Arabidopsis thaliana*, using different populations and experimental conditions (Keurentjes *et al.* 2007; West *et al.* 2007; Cubillos *et al.* 2012; Snoek *et al.* 2012; Lowry *et al.* 2013; Hartanto *et al.* 2020). As an in silico alternative, a prioritization method can help to limit the number of candidate eQTGs for further validation.

Several network-based methods have been used to find eQTGs (e.g. in Keurentjes *et al.* 2007; Jimenez-Gomez *et al.* 2010; Hartanto *et al.* 2020). These methods primarily aim to find master regulator(s) at loci where *trans*-eQTLs for many genes are collocated, known as eQTL hotspots (Breitling *et al.* 2008). In general, these methods utilize a coexpression network built using genes having an eQTL on the hotspot (called *targets*) and genes located in the hotspot (called *candidate eQTGs*). Candidates are then usually prioritized based on a network centrality measure, such as degree centrality (i.e. the number of genes interacting with a candidate) or closeness centrality (i.e. the average path length between a candidate and all other genes) (Serin *et al.* 2016; Hartanto *et al.* 2020). Several candidate eQTGs have been identified in this way, for example, *GIGANTEA* (Keurentjes *et al.* 2007), *ELF3* (Jimenez-Gomez *et al.* 2010), *ICE1*, and *DEWAX* (Hartanto *et al.* 2020). This approach, unfortunately, only works for eQTL hotspots, not for regions that only have a small number of eQTLs. Another limitation is the sole reliance on coexpression data: given the complexity of gene expression regulation, the expression of the regulator is not necessarily correlated to that of its targets, particularly in eukaryotes (Lelli *et al.* 2012; Marbach *et al.* 2012). Therefore, additional data sources should be considered to capture possible interactions between the regulator and its target.

Previously, a machine-learning-based method, QTG-Finder, was developed to prioritize candidate genes for phenotype QTLs in *Arabidopsis* (Lin *et al.* 2019). This method used features derived from various gene properties, such as paralog copy number, gene ontology (GO), and the number of SNPs, to rank the candidate genes in the QTL interval. The model could recall 64% of *Arabidopsis* QTGs when the top 20% ranked genes were considered. Further development of this method led to QTG-Finder2, which used orthology information and allowed for gene prioritization in species with no or few known QTGs (Lin *et al.* 2020). We were curious about the capability of this algorithm to prioritize eQTGs, given that some QTGs are involved in gene expression regulation, for example, *ELF3* (Jimenez-Gomez *et al.* 2010), *ERECTA* (Terpstra *et al.* 2010), *FRI* (Lowry *et al.* 2013), *MAM1* (Jansen *et al.* 2009), and *AOP2* (Jansen *et al.* 2009).

We propose eQTG-Finder, an extended version of QTG-Finder2 for eQTG prioritization, and apply the new algorithm to prioritize eQTGs in *Arabidopsis*. eQTG-Finder contains 12 new features based on protein–protein interaction (PPI), gene structure, and expression variation. Three of these features significantly improve model performance, which is underscored by a feature importance analysis. We demonstrate the efficacy of this algorithm in prioritizing eQTGs using an independent test set. Finally, we use the new model to predict all *Arabidopsis* genes and make these available in our *Arabidopsis* eQTL analysis platform AraQTL (https://www.bioinformatics.nl/AraQTL/)(Nijveen *et al.* 2017) to help identify gene expression regulators.

## Materials and methods

QTG-Finder2 was developed for prioritizing causal phenotype QTL genes (QTGs) in *Arabidopsis* (Lin *et al.* 2020). This algorithm consists of 5,000 Random Forest classifiers (Ho 1998) trained using known QTGs and *Arabidopsis* orthologs of QTGs from other species as positives and other genes as negatives. QTG-Finder2 prioritizes candidate genes based on features generated from polymorphism data, functional annotation, cofunction networks, and paralog copy numbers. Our method extends QTG-Finder2 with new features, and we train the resulting model using the same sets of positive and negative genes. We evaluate the performance in prioritizing candidate causal eQTL genes (eQTGs) in *Arabidopsis*.

### New features

We generate and include 12 new features in addition to the ones already used by QTG-Finder2. These new features are based on PPI, gene expression, and gene/protein structure.

#### PPI feature

Genes can be associated with other genes, for instance, because the encoded proteins participate in the same pathway or are mentioned in the same publication. The number of such interactions a gene has could measure its propensity to be an eQTL causal gene. We generate a network-based feature using *Arabidopsis* PPI data from STRING-DB (Szklarczyk *et al.* 2019). The data were downloaded from the download page of STRING-DB version 11 (https://string-db.org/cgi/download). We only keep high-confident interactions by removing those with STRING scores below 700. We count the number of interactions of each *Arabidopsis* gene as a feature.

#### Gene expression features

We previously showed that different stages of seed germination each have a unique eQTL landscape pointing to stage-specific regulators (Hartanto *et al.* 2020). This indicates that variation in gene expression may help distinguish eQTL causal genes from other (noncausal) genes. We, therefore, generate 7 features based on the average and standard deviation of gene expression across different tissues, accessions, and conditions (control vs. treatments):

##### Tissues

We downloaded RNA-seq data for 9 different tissues (flower, root, male organ, seeds, female organ, stem, leaf, apical meristem, and root meristem) from CoNekT (http://www.evorepro.plant.tools/) (Julca *et al.* 2020). For each gene, the standard deviation is calculated and used as a feature (“SD exp. Across tissues”).

##### Accessions

We used RNA-seq data measured in seedlings of 19 different *Arabidopsis* accessions (Zu-0, Wu-0, Ws-0, Wil-2, Tsu-0, Sf-2, Rsch-4, Po-0, Oy-0, No-0, Mt-0, Ler-0, Kn-0, Hi-0, Edi-0, Ct-1, Col-0, Can-0, and Bur-0). These data are obtained from the *Arabidopsis* RNA-seq Database (http://ipf.sustech.edu.cn/pub/athrna/) (Zhang *et al.* 2020). The average and standard deviation were calculated and used as features (“avg exp. across accessions” and “SD exp. across accessions”).

##### Conditions

From the same database, we collected whole tissue RNA-seq data of the wild-type Col-0 accession. We divided these data into experiments with and without treatments to generate 4 features for average and standard deviation of treatment and control conditions (“avg exp across treatments,” “avg exp. across controls,” “SD exp. across treatments,” and “SD exp. across controls”).

We removed datasets from the *Arabidopsis* RNA-seq Database with a very low total read count and/or many unmapped reads. The list of samples used to generate gene expression features can be found in Supplementary Table 6.

#### Structural features

The structure of causal genes and encoded proteins might differ from the other genes. Therefore, we generate 4 structural features: the numbers of introns, total protein domains, unique protein domains, and splice variants per gene. Data were retrieved from https://www.arabidopsis.org/ (accessed May 2021). The number of introns and splice variants are counted in TAIR10's BLAST datasets. The other 2 features are generated from all.domains.txt by counting each *Arabidopsis* gene's total number of domains and the number of unique domains.

### Hyperparameter tuning

Model evaluation is based on QTG-Finder (Lin *et al.* 2019) and QTG-Finder2 (Lin *et al.* 2020). Given the low number of known eQTGs, we use known QTGs and *Arabidopsis* orthologs of QTGs found in other species as positives and other genes as negatives, similar to QTG-Finder2. We use hyperparameter tuning to determine the best parameter combination (the number of trees, minimal samples split, and maximum number of features) using grid search and assess the area under the curve (AUC) of the receiver-operating characteristic (ROC) curve in an extended version of the 5-fold cross-validation framework. In this framework, the positives are randomly re-split into a training and validation set in a 4:1 ratio iteratively. Next, each set is combined with randomly selected negatives. The ratio of positives and negatives is an optimized hyperparameter. This splitting of positives is done 50 times, and for each positive set random selection of the negatives was conducted 50 times. This extensive procedure (2,500 evaluations) makes that positive cooccurs with all negative at least once with high probability. All machine-learning model training and testing in this study is performed using Python’s scikit-learn library version 1.0.2.

### Selection of candidate eQTL genes and independent validation of model performance

A list of candidate eQTGs in *Arabidopsis* is manually selected from the literature. These genes are categorized as confirmed/strong-candidate, hypothetical, or hypothetical-ortholog. Genes that have been through experimental validation or have strong evidence as eQTG are categorized into the confirmed/strong-candidate group, for example, *GIGANTEA* (Keurentjes *et al.* 2007; Snoek *et al.* 2012). Some confirmed/strong-candidate eQTGs are used as positive in QTG-Finder2, and we remove these from the positive instances to be used as validation genes. Meanwhile, genes that were not experimentally validated but are predicted to play a role as eQTG through *in silico* analysis (e.g. network analysis) are categorized as hypothetical, for example, *ICE1* and *DEWAX* (Hartanto *et al.* 2020). If a gene's ortholog is considered an eQTG in another species, it is categorized as hypothetical-ortholog; for example, *NF-YC4* is found as an eQTG in potatoes (van Muijen *et al.* 2016). In total, this yields 25 candidate eQTGs in *Arabidopsis*: 6 confirmed/strong-candidate, 4 hypothetical, and 15 hypothetical-ortholog genes (Supplementary Table 1). We ensure that these candidates are not used for hyperparameter tuning or cross-validation.

Independent validation is performed using the best combination of parameters (Supplementary Table 5). We train 5,000 Random Forest classifiers using all positives but different sets of negatives, with a positive: negative ratio of 1:200 to approximate the ratio of causal and noncausal genes in real eQTLs. The models are then applied to each candidate eQTG and other genes located within 2 Mbp around it (1-Mbp upstream and 1-Mbp downstream). For these genes, the average probability of being causal is calculated over 5,000 models. These average probabilities are then ranked for prioritization, and the rank is calculated as a performance measure. For example, a rank of 10% indicates that 10% of genes in the eQTL region rank higher than the candidate.

### Feature importance analysis

Feature importance is determined using a leave-one-out analysis. Iteratively, each feature is removed from the dataset, and a model is trained using the reduced dataset. The AUC difference between the full model (with all features) and the reduced model is then calculated and used to indicate the feature importance. In addition, we calculate feature importance for clusters of correlated features. Features are clustered if they have a pairwise Pearson correlation equal to or larger than 0.6. We use the previous cross-validation framework and the best parameters to measure the model performance in this analysis.

### Data analyses

Pairwise Pearson correlation coefficients between features are calculated using the Pandas (version 1.3.5) DataFrame.corr method in Python. Pearson Wilcoxon rank sum test analyzes differences in the median between positive and negative genes for the 12 new features. The test is conducted in R using the base “wilcox.test” function. GO enrichment analysis for the top and bottom 5% predicted causal genes is performed using TopGO in R (Alexa *et al.* 2006) using the algorithm's default “weight01” parameter, which is the mixture of “elim” and “weight” methods. The Python version used for the analyses is 3.8.12, and the R version is 4.0.2.

## Results

The QTG-Finder2 algorithm could rank phenotype QTL causal genes higher than other genes in a cross-validation setting (AUC = 0.81) and recall 80% independent curated causal genes when the top 20% of genes in the QTL are considered (Lin *et al.* 2020). In this study, we extend QTG-Finder2 with a set of new features and evaluate its performance in prioritizing expression QTGs.

### New features improve causal gene prediction performance

**Fig. 1.**
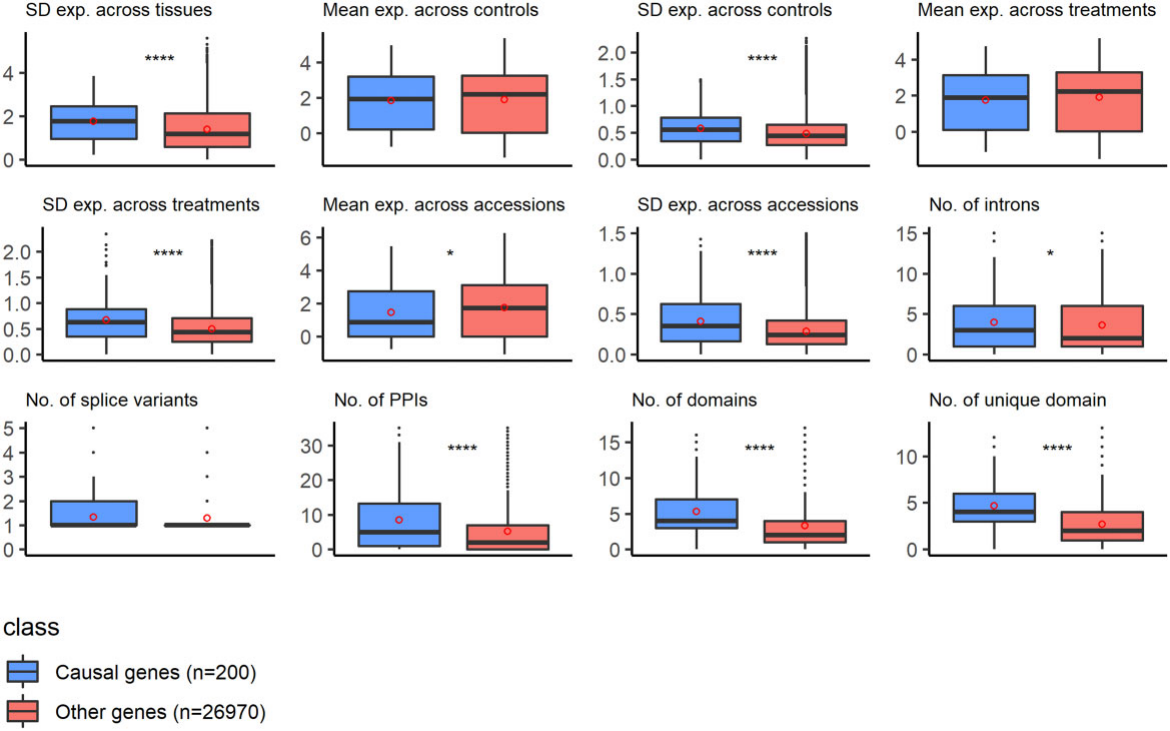
Distribution of 12 new features for known causal genes as the positive class (*n* = 200; 55 known QTGs and 145 orthologs of QTGs from other species) and the remaining genes in the genome as the negative class (*n* = 26,970). Significance of differences in medians was assessed using the Wilcoxon rank sum test (*P ≤ 0.05; ****P ≤ 0.0001). Red dots indicate means. SD, standard deviation; Exp., gene expression.

To improve model performance and better tailor it fit for eQTG prioritization, we added 12 new features based on gene expression, structure, and PPI in the QTG-Finder2 algorithm. Most new features only show a low to moderate correlation with the existing ones (Supplementary Fig. 1), indicating that we add new information to the model. Figure 1 shows feature distributions for the causal genes as the positive class (55 known QTGs and 145 *Arabidopsis* ortholog of QTGs from other species) and the other genes in the genome as the negative class (*n* = 26,970). For most features, the causal genes' median value is significantly different from that of the other genes in the genome (see Supplementary Table 2). The expression of causal genes is more variable than that of other genes. Moreover, causal genes tend to have more and varied protein domains. Causal genes also have slightly more introns than other genes. These differences between the causal genes and the other genes in the genome provide a first indication of potential discriminating features for the machine-learning model. We assess the performance of the model with and without new features using a cross-validation framework.

**Fig. 2.**
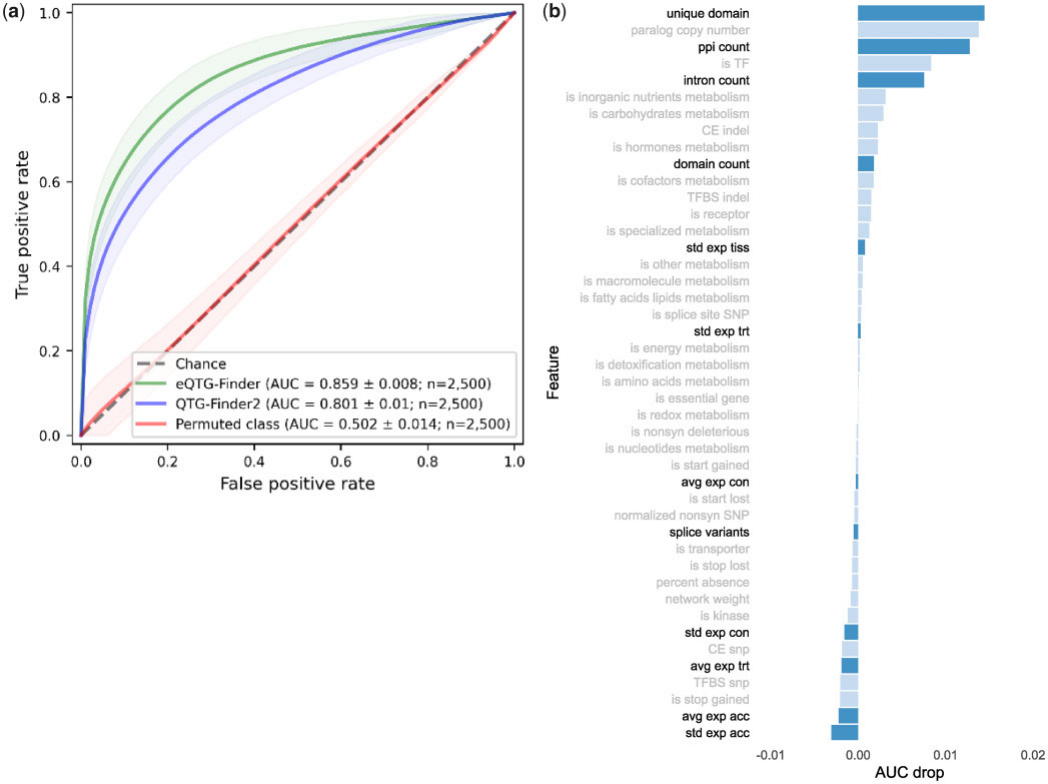
a) AUC of the ROC of the original QTG-Finder2 model (blue) and extended eQTG-Finder model (green), and eQTG-Finder trained with randomized class labels (red) as a control. Transparent areas indicate standard deviations over 2,500 repetitions. b) Feature importance is measured using leave-one-out analysis. A positive AUC drop indicates that the removal of the feature reduces the model's predictive capability. Feature names in bold and with dark blue bars indicate new features. Error bars indicate standard deviations over 2,500 repetitions.

To assess the contribution of new features to the model performance, we compare the AUC of the ROC between the original QTG-Finder2 and the extended model that we labeled eQTG-Finder, and for the extended model with the class labels permutated, as a control (Fig. 2a). The AUC was measured in an extended cross-validation setting over 2,500 different combinations of positive and negative gene sets. The results show that eQTG-Finder (AUC = 0.859 ± 0.008) performs better than QTG-Finder2 (AUC = 0.801 ± 0.01) and the control model (AUC = 0.502 ± 0.014). Adding new features thus allows the model to rank causal genes higher than the other genes. The next section analyzed model performance in prioritizing eQTG using selected candidate eQTGs.

To determine how the new features contribute to causal gene prediction, we calculate feature importance using a leave-one-out approach (Fig. 2b). Each feature is iteratively removed from the dataset, and the reduced model’s performance is compared to that of the model containing all features. The drop in AUC indicates a feature's importance. A positive AUC drop means removing that feature decreases the model's predictive capability. The result shows that 4 of the most important features in the model are the new ones: the number of unique domains, the PPI count, the intron count, and the domain count. However, the large standard deviation for the domain count AUC drop indicates that the contribution of this feature is not consistent over different samples of positive and negative sets.

Some features in the model are highly correlated (Supplementary Fig. 1). When one of these features is removed to calculate feature importance, the reduced model will resort to using these correlated features. As a result, the removed feature might be assigned lower importance than it should have in the model (Gregorutti *et al.* 2017). To avoid this, we calculated feature importance for clusters of features. The result (Supplementary Fig. 3) shows a slight change in the importance of some features, for example, “network weight” is now among the top important features since it is correlated with “ppi count.”

### eQTG-Finder ranks most strong eQTG candidates better than QTG-Finder2

To evaluate eQTG prioritization performance, we again train the original QTG-Finder2 and the extended eQTG-Finder model and use them to rank selected potential eQTGs (Supplementary Table 1). Models are trained using all positives (known QTGs and *Arabidopsis* ortholog QTGs from other species). We repeated the training 5,000 times with different negative samples to select each negative gene at least once in training with high probability. These models rank each of the 25 potential eQTGs with their surrounding genes within a 2-Mbp window as a hypothetical eQTL region. These potential eQTGs are selected manually from the literature and grouped based on the evidence of being causal eQTL genes (see *Materials and Methods* for detail). Gene ranking is based on the average probability of a gene being causal, as predicted by the 5,000 models. We use the rank to indicate the percentage of genes on the eQTL with higher ranks than the gene of interest (i.e. a rank of 10% indicates that 10% of genes in the eQTL region rank higher than the gene of interest). We predefine cutoffs of 5%, 10%, and 20%, in each of which we compare recall between QTG-Finder2 and eQTG-Finder. These recalls for different cutoffs can be used by researchers to decide the proportion of top prioritized genes for further experimental validation.

**Fig. 3.**
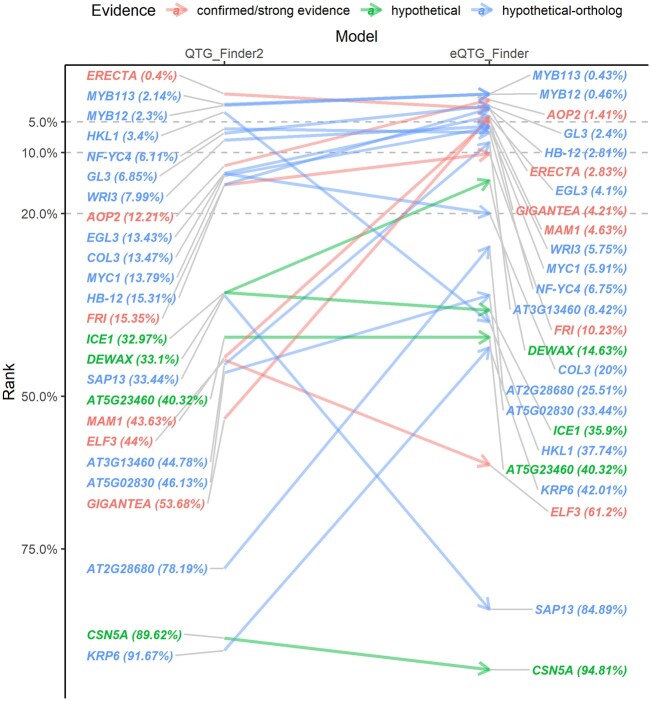
Rank comparison of 16 candidate eQTGs using the model with new features (eQTG-Finder) and the original model (QTG-Finder2).

The QTG-Finder2 model recalls 16%, 28%, and 52% of eQTG candidates if the top 5%, 10%, and 20% ranked genes are considered (Fig. 3). With added features, eQTG-Finder ranks eQTGs slightly better with percentages of 36%, 52%, and 64% respectively. The eQTGs vary in their evidence of being causal genes (see *Materials and Methods*). Four out of 6 strong eQTG candidates (*AOP2*, *ERECTA*, *GIGANTEA*, and *MAM1*) rank within the top 5% by eQTG-Finder compared to only one (*ERECTA*) by QTG-Finder2. The other 2 strong candidates, *FRI* and *ELF3*, were ranked at 10.2% and 61.2% by eQTG-Finder. The ranks of 16 genes are improved by eQTG-Finder, 8 are worse, and 1 stays the same (Supplementary Table 3). The rank of 4 out of 6 strong eQTG candidates improves, with *GIGANTEA* one of the most drastic improvements, moving from 53.7% to 4.2%. On the other hand, the rank of *ERECTA* drops (0.4–2.8%) but remains in the top 5%. Both models rank another strong eQTG candidate *ELF3* poorly (at 44% by QTG-Finder2 and 61.2% by eQTG-Finder). As the number of strong eQTG candidates is limited, we also show the prioritization of hypothetical and hypothetical orthologs eQTGs. Even though the improvement was not as large as for the strong eQTG candidates, eQTG-Finder still ranks most of the hypothetical and hypothetical-ortholog eQTGs in the top 10%.

Despite the decent overall performance in candidate eQTGs prioritization, we notice that eQTG-Finder performance in prioritizing phenotype QTGs is still inconsistent. Using the initial independent validation set, only 7 out of 11 QTGs are ranked within the top 20% by eQTG-Finder, compared to 9 by QTG-Finder2 (Supplementary Fig. 2).

**Fig. 4.**
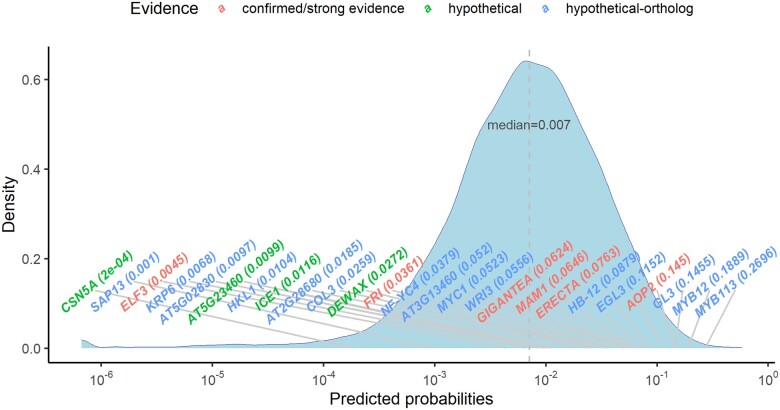
The density plot of probabilities of being causal predicted by eQTG-Finder for all *Arabidopsis* genes. Text labels point to the probability of the gene in the plot. The *x*-axis is on a log_10_ scale.

To get an overview of eQTG-Finder predictions, we inspect the distribution of the average predicted probability of being causal for all *Arabidopsis* genes (Fig. 4). This skewed toward a low value, with a median value of 0.007 (note that the *x*-axis of Fig. 4 is on a log_10_ scale). Twenty-one of the 25 genes in the validation set have a predicted probability higher than the median. *ELF3* (probability = 0.0045) is the only strong eQTG candidate with a predicted probability lower than the median.

A GO enrichment analysis shows that the top 5% genes in the distribution are significantly enriched (false discovery rate *P*-value <0.05) for 67 GO terms (Supplementary Table 4), most of which are related to response to abiotic and biotic stresses, such as “defense response to bacterium,” “defense response to fungus,” and “response to wounding.” The term “regulation of transcription” is also enriched, suggesting that transcription factors are likely to be causal, consistent with the feature importance analysis result where “is_TF” is among the most important features. Meanwhile, the bottom 5% are not enriched for any term.

### eQTG-Finder is available in AraQTL to support new hypotheses on the gene expression regulation

**Fig. 5.**
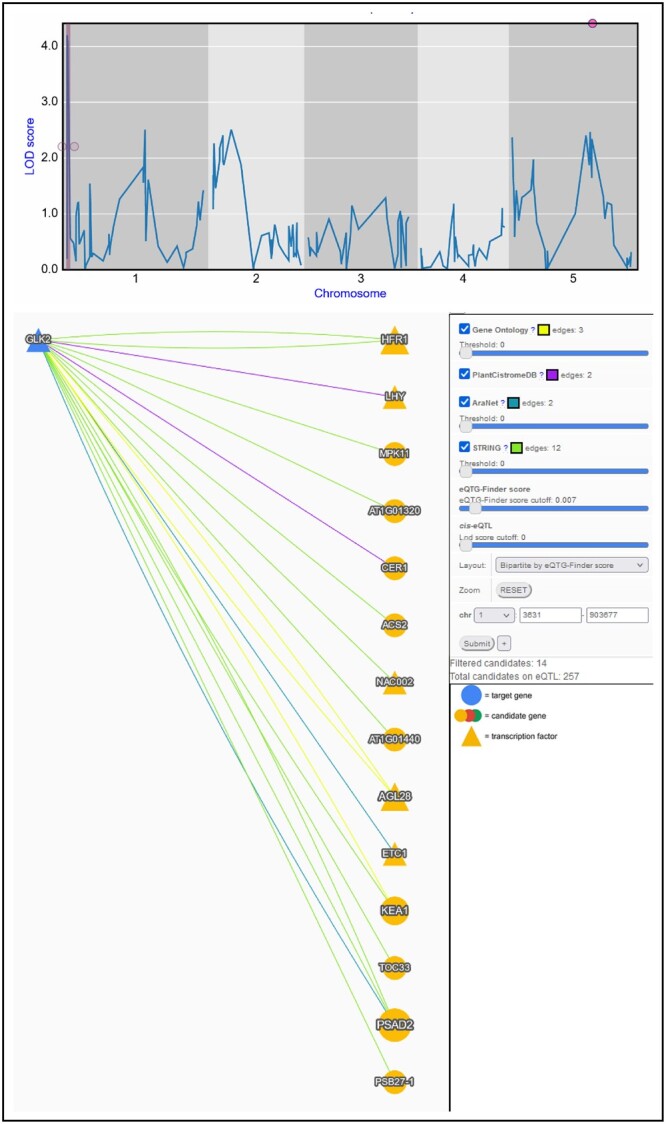
Prioritization of *GLK2* regulator using the eQTG-Finder score in AraQTL. (Top) eQTL profile of *GLK2* from the Lowry *et al.* (2013) experiment. The eQTL region on chromosome 1 (shaded in pink) pinpoints the location of potential *GLK2* regulator(s). (Bottom) Prior knowledge network connecting *GLK2* (blue node) with candidate regulators (yellow nodes) based on prior knowledge data. Here, the eQTG-Finder score is used to order candidates based on their probability of being causal.

To make eQTG-Finder results easily accessible for researchers, we include predicted probabilities of causality (herewith referred to as eQTG-Finder score) for all *Arabidopsis* genes in AraQTL, our *Arabidopsis* eQTL data workbench (Nijveen *et al.* 2017). Prioritizing genes using QTG-Finder2 is not straightforward as it requires users to prepare a list of candidate genes and command-line usage skills. Integrating the eQTG-Finder score in AraQTL facilitates users to interactively identify gene expression regulators. For example, we here discuss a case on predicting a new potential regulator for *GLK2* using the eQTG-Finder score and other interaction evidence in AraQTL. *GLK2* is a GARP nuclear transcription factor involved in light-controlled signaling (Waters *et al.* 2009). Liu *et al.* (2022) recently found that *HY5* is the regulator of *GLK2* based on the fact that *HY5* is a well-known regulatory switch for light signaling in literature. The same conclusion can also be derived using the Serin *et al.* (manuscript in preparation) eQTL experiment and prior knowledge data in AraQTL. Another approach to finding potential regulators of *GLK2* can be made in AraQTL using the eQTG-Finder score. In a Kas × Tsu eQTL experiment on leaf tissue (Lowry *et al.* 2013), *GLK2* has an eQTL on the beginning of chromosome 1, indicating the location of the potential regulator(s) (Fig. 5, top). As many as 257 candidate regulatory genes are present in the eQTL (Fig. 5, bottom). We can filter out weak candidates by constructing a network of *GLK2* connected to its potential regulators on the eQTL based on prior knowledge, such as PPI and gene annotation (Hartanto *et al.*, manuscript in preparation). Here, we threshold the eQTG-Finder score to remove weak candidates. Moreover, eQTG-Finder can prioritize the remaining 14 genes by selecting the “Bipartite by eQTG-Finder score” network layout and ordering genes by their score. The result suggests some promising *GLK2* regulator candidates ranked at the top, for example, a transcription factor *LHY* in second place*.* Until now, *LHY* has not been reported to regulate *GLK2.* However, this gene is a promising *GLK2* regulator candidate as the network shows that it has a transcription factor binding site(s) on the *GLK2* promoter (O'Malley *et al.* 2016). Moreover, *LHY* is involved in light signaling (Kim *et al.* 2003; Joo *et al.* 2017). This example suggests that integrating the eQTG-Finder score in AraQTL can help infer new regulatory interactions.

## Discussion

The concept of genetical genomics was first coined 2 decades ago (Jansen and Nap 2001), and numerous *Arabidopsis* eQTL data sets have been published since then (Nijveen *et al.* 2017). The aim of genetical genomics is to pinpoint genomic regions associated with gene expression variation (eQTL) and ultimately unravel genes involved in expression regulation. However, identifying causal genes (eQTGs) is difficult because of the often large genomic regions they span, regularly harboring dozens or even hundreds of candidates. The regions can be narrowed down by experimental fine-mapping (Eshed and Zamir 1995), and the remaining candidate genes can then be validated using functional genomics methods (e.g. using CRISPR-Cas9-mediated deletions as in Evans and Andersen 2020). However, performing these experiments for thousands of eQTLs is very costly. Using genomics and annotation data, a computational prioritization method can help identify candidate eQTGs. This study extends an existing machine-learning algorithm, QTG-Finder2, to address this issue and evaluates its performance in prioritizing eQTG. eQTG-Finder outperforms QTG-Finder2 in distinguishing positive causal genes from the other genes in the genome based on a cross-validation setting (Fig. 2a). Moreover, eQTG-Finder prioritizes most eQTGs in eQTLs better than QTG-Finder2 in an independent validation test (Fig. 3). We make eQTG-Finder scores available in AraQTL to help researchers interactively identify key regulators.

The key improvement of eQTG-Finder lies in the inclusion of 12 new features based on gene expression, structure, and interactions. Given the complexity of the resulting model, it is not straightforward to assess how these features improve eQTG-Finder in gene prioritization (Petch *et al.* 2022). We calculated the contribution of each feature in the model using a leave-one-out feature importance analysis (see *Materials and Methods*). This showed that the number of unique protein domains, the number of PPI and the number of introns are in the top 5 most contributing features in the model. We showed that known causal genes tend to have more domains, PPI partners, and introns than other genes (Fig. 1). These new features may provide insight into what distinguishes causal and noncausal genes. For instance, since protein domains determine protein functions (Enright and Ouzounis 2001; Vogel *et al.* 2004), the presence of multiple domains in a causal gene could indicate involvement in a wide range of biological functions. The diverse functions of causal genes could also be reflected in their larger number of PPI partners than noncausal as genes perform their function in concert with other genes (Ito *et al.* 2001). The number of introns reflects the number of exons in a gene. Several studies demonstrated that exons play a role in the evolution of domain architectures through exon-shuffling, leading to new combinations of domains with new functions.

Variation in phenotype can be traced back to variation in gene expression (Skelly *et al.* 2009; Albert and Kruglyak 2015). For this reason, we included features based on the standard deviation (SD) of gene expression across different *Arabidopsis* accessions, tissues, and conditions. Even though the medians between causal and other genes are significantly different (Fig. 1), features based on SD of expression have low importance in the model (Fig. 2a). A possible explanation for this could be that features based on expression are correlated (Supplementary Fig. 1) and, therefore, their importance is underestimated (Gregorutti *et al.* 2017). We, therefore, removed all of these correlated features and re-calculated the feature importance. The feature importance, however, remains the same. Nevertheless, we do not have evidence that these features negatively affect the prediction performance; hence, we kept them in the model.eQTG-Finder uses known QTGs (i.e. causal genes for a phenotype QTL) as positive instances for model training because of the limited number of known eQTGs. A recent finding in humans showed that *cis*-eQTLs and GWAS genes are different due to the detection bias of the assays (Mostafavi *et al.* 2022). This detection bias could also hold for trans-eQTL and phenotype QTL genes in *Arabidopsis*. However, we argue that QTGs are still relevant for prioritizing eQTG since variation at the molecular level (e.g. in gene expression, metabolite, or protein level) can be propagated and cause variation at higher phenotypic levels (Fu *et al.* 2009; Civelek and Lusis 2014). For example, genetic variations in *AOP2* and *MAM1* cause *cis*-eQTLs for gene expression and metabolite QTLs for aliphatic glucosinolate biosynthesis, which confer insect resistance in *Arabidopsis* (Wentzell *et al.* 2007; Jansen *et al.* 2009). Both genes were prioritized in the top 5% by eQTG-Finder. This result suggests that eQTG-Finder can identify QTLs for other molecular phenotypes, including metabolite and protein.

A lack of model interpretability may hamper a user’s comprehensive evaluation and assessment of the prioritization results. Regardless of the good performance, it is difficult to precisely understand how eQTG-Finder classifies certain genes as causal and others as noncausal, a typical issue for a complex model like Random Forest (Petch *et al.* 2022). Instead, in AraQTL, we provide additional sources of evidence to support the eQTG-Finder prioritization results (Hartanto *et al.*, unpublished). For example, eQTG-Finder prioritizes transcription factor *LHY* as the regulator of *GLK2* (Fig. 5). The network visualization in AraQTL showed that *LHY* is connected to *GLK2* by transcription factor binding site evidence, indicating that *LHY* may bind to the *GLK2* promoter and modulate its expression. Incorporating eQTG-Finder in the AraQTL web interface facilitates researchers to identify key regulators for genes of interest without the need for computational skills.

In the independent validation, some eQTG candidates were ranked poorly by eQTL-Finder (Fig. 3). Low-ranked assumed eQTG genes from the hypothetical and hypothetical-orthologs groups might not be actual eQTGs; however, the strong eQTG candidate ELF3 was also ranked poorly by both eQTG-Finder (61.2%) and QTG-Finder (44%). *ELF3* encodes a nuclear protein and was demonstrated to regulate gene expression leading to shade-avoidance response (Jimenez-Gomez *et al.* 2010). The complexity of the eQTG-Finder algorithm makes it difficult to dissect the prediction for *ELF3*. We investigated 2 of the most important features and noticed that this gene only has 1 identified protein domain and 1 paralog copy number, which is lower than the median values of causal genes (4 and 17, respectively).

We observed that eQTG-Finder prioritization of candidate QTGs in independent validation was slightly worse compared to QTG-Finder2 (Supplementary Fig. 2), despite its better performance in cross-validation (Fig. 2b). The new expression-based features might bias eQTG-Finder toward prioritizing eQTGs compared to QTGs, but the complexity of the model makes it difficult to learn exactly how these features affect prioritization. Moreover, the number of 11 candidates we used for validation is too low to allow a very precise assessment of the general performance of eQTG-Finder in prioritizing QTGs.

Likely, some features associated with eQTG are still missing in our model or underrepresented in our set of positive instances. Since the regulator-target relationship is specific, we expect that features representing gene–gene/PPI [e.g. STRING scores (Szklarczyk *et al.* 2019), transcription factor binding sites (Tian *et al.* 2020), and GO semantic similarity (Yu 2020)] are relevant for prioritizing eQTG. Including these would shift the prioritization of generic eQTGs based on gene properties to the prioritization of eQTGs for a specific target using features based on gene-pair relationships. This is similar to the approaches of Wong *et al.* (2004) and Pandey *et al.* (2010), who predicted genetic interaction using gene pair relationships in yeast. The number of positive examples (i.e. confirmed eQTG-target pairs) is currently too small to properly train such a model for *Arabidopsis*. However, as data regarding genetic regulation is steadily increasing, we are optimistic that this strategy will be possible in the future.

## Supplementary Material

jkac255_Supplementary_Table1_2_4_5

jkac255_Supplementary_Figure_S1

jkac255_Supplementary_Figure_S2

jkac255_Supplementary_Figure_S3

jkac255_Supplementary_Table3

jkac255_Supplementary_Table6

## Data Availability

The code and data for the analysis and visualization are available at the Wageningen University GitLab repository (https://git.wur.nl/harta003/eqtg-finder). eQTG-Finder prioritization is available at AraQTL (https://www.bioinformatics.nl/AraQTL/; Nijveen *et al.* 2017). Supplemental material is available at G3 online.
