# Supplementary figures and images for: Prioritizing candidate eQTL causal genes in Arabidopsis using RANDOM FORESTS
Source: G3 (Bethesda). 2022 Sep 23;12(11):jkac255. doi: 10.1093/g3journal/jkac255 (PMC9635658; doi:10.1093/g3journal/jkac255)

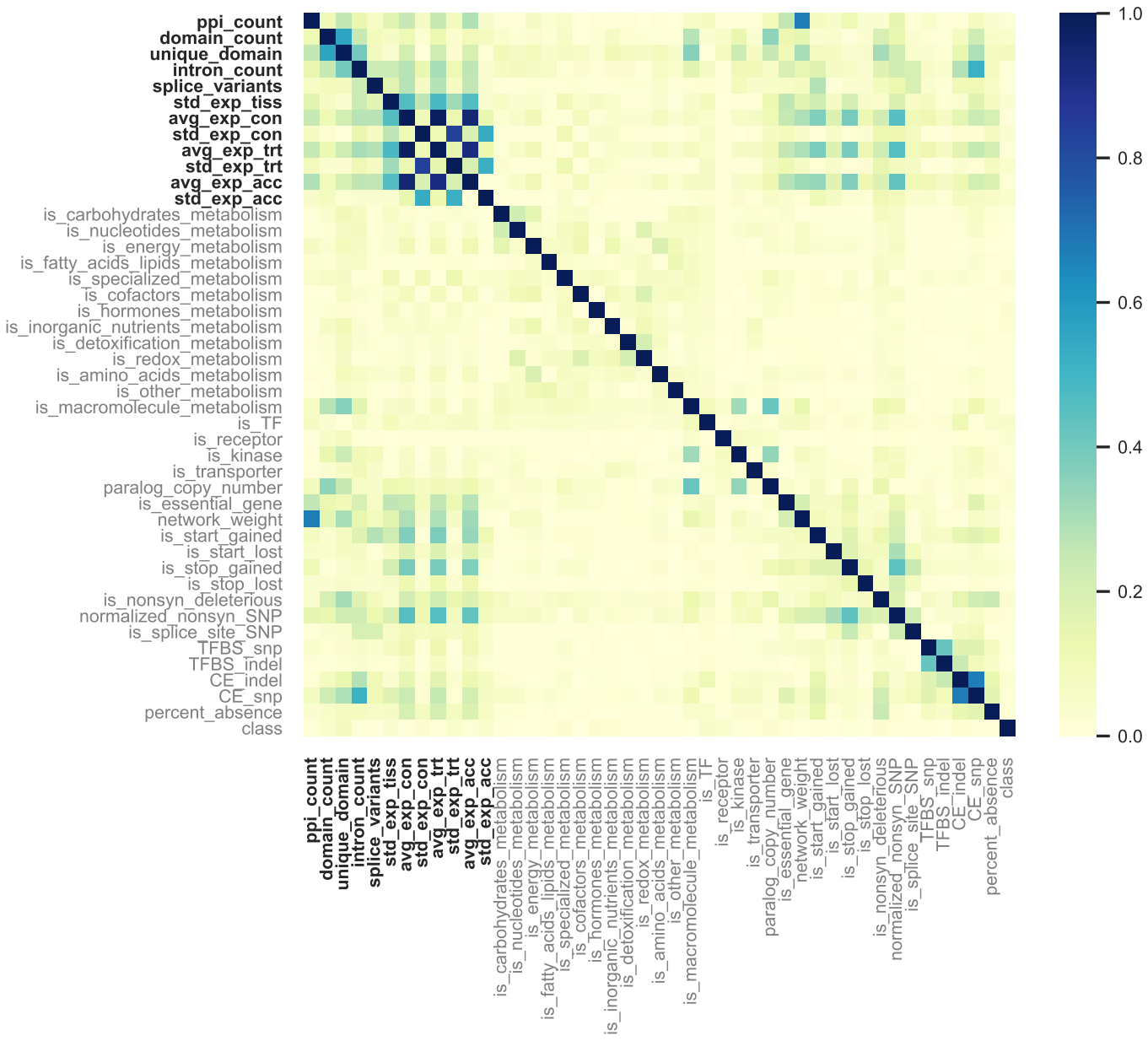

Supplement: jkac255_Supplementary_Figure_S1 [file jkac255_supplementary_figure_s1.pdf]

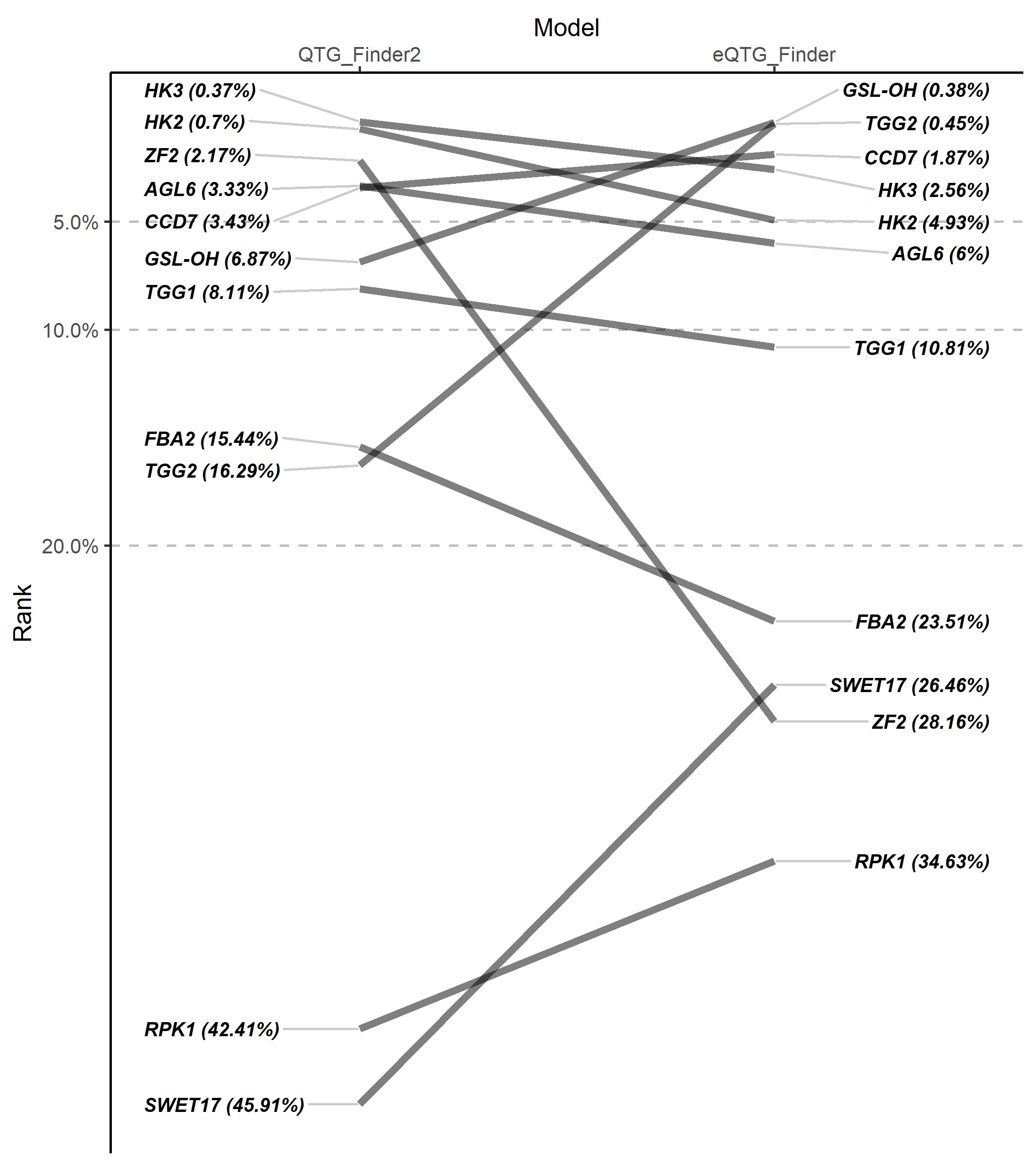

Supplement: jkac255_Supplementary_Figure_S2 [file jkac255_supplementary_figure_s2.jpeg]

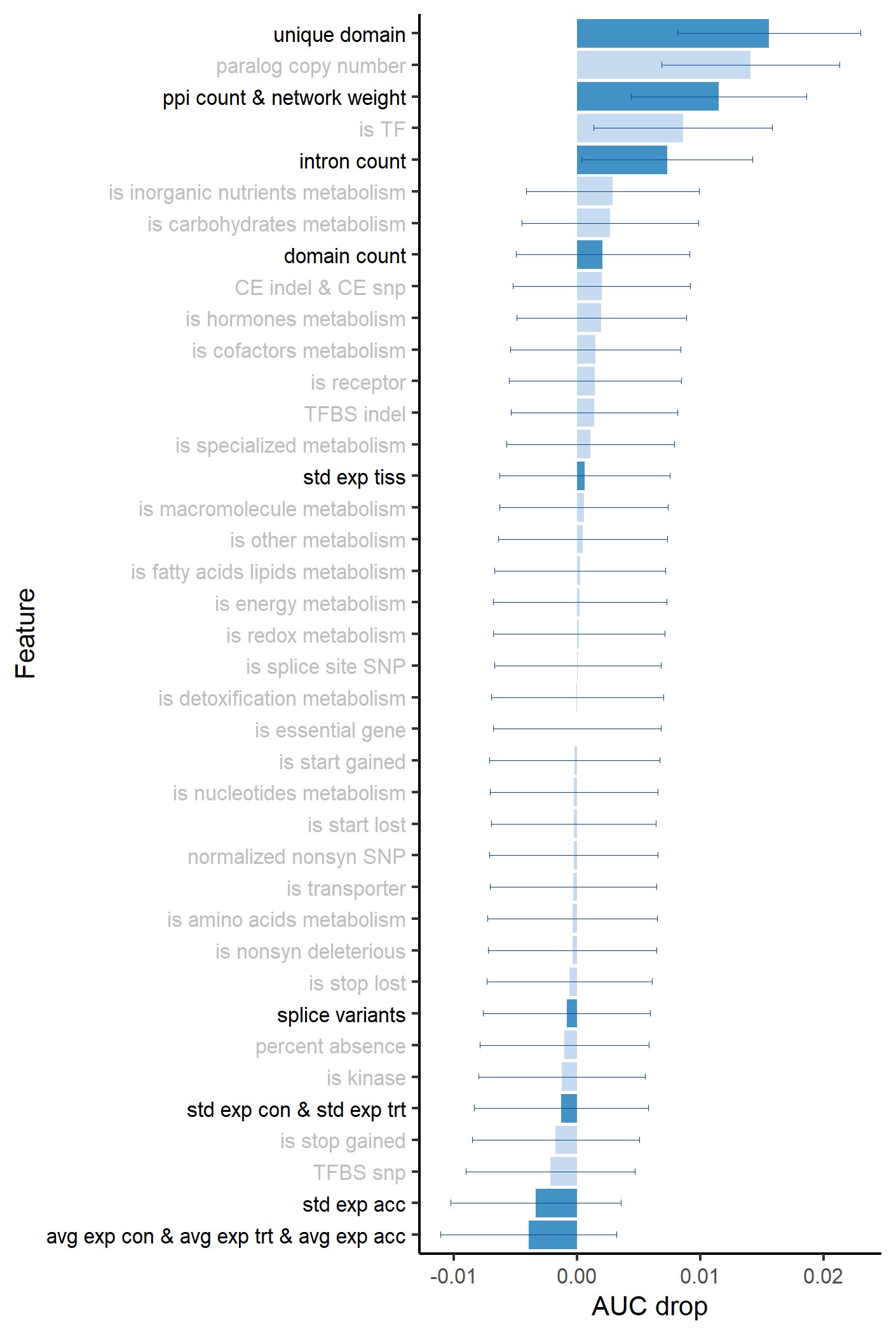

Supplement: jkac255_Supplementary_Figure_S3 [file jkac255_supplementary_figure_s3.jpeg]
